# Self-Reported Patterns of Use of Alcohol and Drugs After Suicide Bereavement and Other Sudden Losses: A Mixed Methods Study of 1,854 Young Bereaved Adults in the UK

**DOI:** 10.3389/fpsyg.2020.01024

**Published:** 2020-05-20

**Authors:** Alexandra Pitman, Fiona Stevenson, Michael King, David Osborn

**Affiliations:** ^1^UCL Division of Psychiatry, University College London, London, United Kingdom; ^2^Camden and Islington National Health Service (NHS) Foundation Trust, London, United Kingdom; ^3^UCL Department of Primary Care and Population Health, University College London, London, United Kingdom

**Keywords:** alcohol, drugs, substance misuse, bereavement, grief, content analysis, qualitative methods

## Abstract

**Background:**

Bereavement, particularly by suicide, is associated with an excess risk of mortality and of physical and psychological morbidity. Use of alcohol as a coping mechanism is suggested as a contributing factor. However, studies describing substance use after bereavement rely on diagnostic data, lacking a more fine-grained understanding of patterns of substance use when grieving. We aimed to use mixed methods to compare patterns of substance use after bereavement by suicide and other sudden deaths among young adults in the UK.

**Methods:**

Using an online survey throughout 37 UK higher education institutions we collected free text responses from 1,854 young adults who had experienced sudden bereavement. We conducted content analysis of free text responses to an open question about patterns of alcohol and drug use following the bereavement, measuring frequencies of coded categories. Collapsing these categories into binary outcomes reflecting increased use of alcohol or drugs, we used multivariable logistic regression to quantify the associations between mode of bereavement and increased post-bereavement substance use.

**Results:**

Of 1,854 eligible respondents, 353 reported bereavement by suicide, 395 by accidental death, and 1,106 by sudden natural causes. The majority of the sample reported no increase in their use of alcohol (58%) or unprescribed drugs (85%) after the bereavement. Overall 33% had increased their alcohol use at some point after the bereavement, whilst 12% had increased their use of drugs. People bereaved by suicide were significantly more likely to describe an increase in substance use (adjusted OR = 1.29; 95% CI = 1.00–1.66; *p* = 0.049) than people bereaved by sudden natural causes, as were people bereaved by non-suicide unnatural deaths (adjusted OR = 1.32; 95% CI = 1.03–1.68; *p* = 0.026).

**Conclusion:**

Just under half of young UK adults who experience sudden bereavement increase their alcohol use afterwards, and very few increase their use of drugs. People bereaved by suicide or non-suicide unnatural deaths may be more likely than people bereaved by sudden natural causes to use substances as part of the grieving process, and may have a greater need for monitoring of potential harms. Understanding the reasons for substance use will help primary care and bereavement practitioners screen and address needs appropriately.

## Introduction

Although grief is a normal response to loss, there is evidence that people who lose a close contact to suicide or other violent causes of death have an increased risk of complicated grief (Lobb et al., 2010), and are more likely to perceive stigma and shame related to the loss (Pitman et al., 2014). This is likely to be related to the violent or sudden nature of the loss, its degree of unexpectedness, and the lack of anticipatory grief. Among people who experience suicide bereavement, complicated grief is associated with risk of suicidal ideation (Mitchell et al., 2005), and there is clear evidence of an increased risk of suicide, psychiatric disorder, and physical health problems after suicide loss (Erlangsen et al., 2017). The burden of grief in people bereaved by sudden unnatural causes is therefore potentially heavier than for people bereaved by natural or anticipated causes, yet we know little about coping mechanisms in this group. We also remain unclear about the explanations for the excess risk of mortality and physical health problems in people bereaved by suicide (Pitman et al., 2014; Erlangsen et al., 2017).

Worden’s theory of grief suggests that bereaved people face a series of tasks, including accepting the reality of the loss, processing the pain of grief, and learning to adjust to a world without the deceased (Worden, 2018). Clinically we observe that some bereaved people use alcohol to help cope with these tasks (Stroebe et al., 2007), and there is evidence to support an increased risk of alcohol disorders after stressful life events (Keyes et al., 2011).

Data from a United States household survey data show that the unexpected death of a loved one was cited as respondents’ most common traumatic experience, and was also most likely to be rated as their worst traumatic experience (Keyes et al., 2014). This same study demonstrated an association between unexpected bereavement and a range of psychiatric disorders across the life course, most commonly alcohol use disorder (36%), particularly in older age groups (Keyes et al., 2014). There is evidence to support increased alcohol use after bereavement in older adults (Byrne et al., 1999; Stahl and Schulz, 2014), and young adults (Hamdan et al., 2013), particularly men (Pilling et al., 2012). Use of alcohol to cope with emotional pain may be particularly characteristic of men (Cleary, 2012), including after bereavement (Creighton et al., 2015). However, despite being used as a coping mechanism, it may even exacerbate their psychological distress (Cleary, 2012).

These clinical and research findings identify alcohol misuse as a potential explanation for the association between suicide bereavement and adverse physical health, mental health and mortality outcomes, although there is little direct evidence to support this hypothesis (Stroebe et al., 2007). Instead, indirect support for this theory arises from the well-established association between alcohol misuse and poor physical and mental health, and with suicide (Connor et al., 2016). Finnish data show that risk of mortality due to violent causes is doubled in the first week after widowhood, and suicide mortality is increased over the first 5 years (Kaprio et al., 1987). Substance misuse is a risk factor for both accidental death and suicide (Bohnert et al., 2010). There is also evidence that mortality risk from alcohol-related causes is elevated after bereavement (Martikainen and Valkonen, 1996). However, few studies have investigated changes in alcohol use after suicide bereavement compared with bereavement by other causes, and none have tested whether substance misuse mediates risk of suicide after suicide bereavement.

The majority of epidemiological studies exploring changes in use of alcohol or drugs after bereavement have relied on diagnostic criteria for alcohol dependence or misuse. A study based on diagnostic interviews with young people found significantly higher rates of current alcohol or substance misuse disorder 21 months after parental loss due to suicide compared with bereavement by accidental death, but was not adjusted for pre-loss substance misuse (Brent et al., 2009). Population-based analyses using population registries have reliable means of adjusting for pre-loss psychopathology, but rely on individuals presenting to services and formal diagnosis being recorded. Such work has established that the risk of alcohol and drug misuse disorders is increased in people bereaved by suicide compared with non-bereaved controls (Erlangsen et al., 2017), but not when compared with people bereaved by other causes (Bolton et al., 2013; Erlangsen et al., 2017). To fully understand the range of drug and alcohol-related behaviors that might change after suicide compared with other losses, it is therefore important to go beyond diagnostic criteria and use alternative data collection approaches to gain a more in-depth understanding of this phenomenon.

In this study we aimed to use mixed methods in order to gain a better understanding of any changes in patterns of use after suicide bereavement compared with other sudden unexpected deaths. We were interested in self-reported patterns rather than formal diagnostic classification systems for substance use (World Health Organisation [WHO], 1992; American Psychiatric Association [APA], 1994) or alcohol use screening tests (Rumpf et al., 2002). This was to gain a sense of the wider role that drugs or alcohol played in bereaved people’s lives, how this relates to their social functioning or physical and mental health, and any motivations for changes in use. Our first objective was to use content analysis of qualitative accounts to gain an understanding of how substance use might be affected by sudden loss, and to investigate whether patterns differ by type of bereavement. Our second objective was to use the results of our content analysis to test the hypothesis that people bereaved by suicide would be more likely to describe increased substance use after bereavement than people bereaved by sudden natural causes of death. We also wished to test whether people bereaved by non-suicide unnatural causes of death would be more likely to describe increased use of substances than those bereaved by sudden natural causes of death.

## Materials and Methods

### Study Design and Participants

We used email sampling and a closed online survey to collect cross-sectional data on young adults working or studying at British higher education institutions (HEIs). This sampling frame was judged to be the most accessible and low-cost means of recruiting a hard-to-reach population of young adults (Pitman et al., 2015), minimizing the biases involved in using a help-seeking sample. Recruitment has been described in detail elsewhere (Pitman et al., 2016). Briefly, in 2010 we invited all 164 HEIs in the UK at that time to participate in an online survey, following-up non-responding HEIs to encourage broad socio-economic and geographic representation. Over 20% of HEIs (37/164) agreed to take part, providing an estimated sampling frame of 659,572 staff and students (a minority of whom were assumed to have experienced sudden bereavement). All participants were invited to take part in a survey of “the impact of sudden bereavement on young adults.” The majority of participating HEIs agreed to send an individual email invitation to each staff and student member, as per study protocol. For reasons of sensitivity, 10 HEIs modified this strategy, for example by sending the study to students and not staff, or using differing combinations of their weekly news digest email or intranet advertisement for staff or students.

Inclusion criteria were as follows: people aged 18–40 who had experienced sudden bereavement of a close friend or relative after 10 years of age. This age range was chosen to reflect an under-researched group of great interest in suicide prevention policy. Early childhood bereavements (prior to age 10) were excluded to reduce recall bias and capture adult cognitive processing of a life event. We used the age of 10 as this was the threshold for criminal responsibility in England and Wales at the time of sampling and therefore represented the threshold for adult cognition. Sudden bereavement was defined as “a death that could not have been predicted at that time and which occurred suddenly or within a matter of days.” Type of bereavement was sub-classified by self-report as: bereavement by suicide, bereavement by sudden natural causes (for example cardiac arrest), and bereavement by sudden unnatural causes (for example accidental death). In the case of exposures to more than one mode of sudden bereavement, we applied a hierarchy such that all those bereaved by suicide were classified as such, regardless of other exposures. Those bereaved by deaths due to sudden natural causes and those due to sudden unnatural causes were asked to relate their responses to whichever person they had felt closest to, and exposure status was classified accordingly. For people bereaved by more than one suicide, respondents were asked to relate their responses to the person they had felt closest to.

### Procedures

Each email invitation contained a link to the online survey. This questionnaire (Pitman et al., 2016). was designed in consultation with a group of young bereaved adults and bereavement counselors, who identified important domains to cover in relation to the impact of bereavement and appropriate wording. It was piloted in an open online survey for individuals accessing support from four national voluntary sector organizations providing bereavement support (Cruse Bereavement Care, Samaritans, Survivors of Bereavement by Suicide, Widowed by Suicide).

The first 120 questions elicited quantitative data on socio-demographic and clinical characteristics (Part 1). These were followed by 20 open questions eliciting free text qualitative data (Part 2) on specific domains affected by the bereavement, such as relationships, spirituality, and finances. Part 2 questions were worded to be non-leading and neutral (i.e., not assuming only negative outcomes of bereavement), and were derived from the literature describing lived experience (Wertheimer, 2001; Simone, 2008) and input from the consultation group. There was no upper word limit, and respondents were invited to give as much or little detail as they wished, or to skip the question if it did not apply to them.

The question relating to use of drugs and alcohol was worded as follows: “*In what way, if any, has the bereavement affected your drinking habits or your use of unprescribed drugs? (Unprescribed drugs include illicit drugs as well as medications used above their prescribed limits).”* To avoid ambiguity in phrasing this question, we chose to use the term unprescribed drugs to cover use of illegal drugs, legal highs, over-the-counter drugs, or prescribed drugs used above advised limits.

### Ethics

The study was approved by the UCL Research Ethics Committee in 2010 (ref: 1975/002). The participant information sheet indicated that the study was being conducted by a research team at UCL, that the results would be analyzed and compiled into a publically available report, and that no individual respondent would be identifiable from this information. All participants provided online informed consent by ticking a box to indicate they had read the participant information leaflet and consent form and agreed that the anonymized results would be used for research purposes.

### Content Analysis

Given the nature and quantity of the data, we chose the approach of content analysis to analyse large volumes of brief free text responses to this question. This is a qualitative research method used to interpret the content of text data through a systematic classification process involving coding and identifying themes (Krippendorff, 2004). In conventional content analysis, the researcher does not start with preconceived ideas about what kinds of codes or categories of codes will be found but uses the data to drive the coding (Hsieh and Shannon, 2005). However, we aimed to use directed content analysis to capture perceived increases or decreases in substance use, as well as perceptions of no change. This approach allowed us to measure the frequency of these codes, and link them to quantitative variables such as exposure, socio-demographic characteristics and time since loss (Ritchie et al., 2013). We deliberately avoiding attempting diagnostic categorization or estimates of the quantity of substances used due to the inherent difficulties using an online survey. The analytic team included a medical sociologist (FS) and clinical academic psychiatrists (AP, MK, DO) to balance clinical and non-clinical perspectives.

#### Qualitative Component of Content Analysis

We imported online responses to the question on substance use into Microsoft Excel, which allowed us to process and code large volumes of relatively brief free text data. We coded free text responses to create a set of codes corresponding to respondents’ perceptions of any changes in patterns of substance use. After familiarization with the first 100 data lines, the first two authors noted that respondents often described any change in their habits in terms of perceptions of helpfulness or harms. They developed an initial coding framework of five categories, which was agreed by all co-authors for face validity. This was as follows:

1. no change
2. Increased use – perceived as harmful.
3. Increased use – perceived as helpful.
4. Decreased use – perceived as harmful.
5. Decreased use – perceived as helpful.

The familiarization process also resulted in a team decision to separate out three separate substances used (alcohol, unprescribed drugs, and cigarettes) given that reported patterns of use varied by substance. Cigarette use had not been specifically inquired about in the survey question, but as some respondents included information about smoking patterns in their responses, this was considered as a separate entity.

The first author then proceeded to code the first 500 responses, further elaborating the set of codes to create new categories and sub-categories of existing codes. These sub-categories reflected temporal changes in patterns of substance use (for example transient increases) as well as perceptions of harm or benefit. This coding was conducted in collaboration with the second author (FS) rather than independently, and involved discussions within the wider team. After coding 500 of responses the coding framework was expanded as follows:

1. No change in substance use.
2. Brief temporary increase (within the week of the death) but then resumed pre-loss pattern of use.
3. Stopped.
4. Reduced.
5. Increased (unclear if perceived as helpful or harmful).
6. Increased (perceived as helpful).
7. Increased (perceived as harmful).
8. Increased (unclear if perceived as helpful or harmful) but then resumed pre-loss pattern of use.
9. Increased (perceived as helpful) but then resumed pre-loss pattern of use.
10. Increased (unclear if perceived as helpful or harmful) but then stopped.
11. Unable to classify.

Responses describing the appropriate use of prescribed medication (for example “*Been prescribed citalopram for depression for the past 3 years*”) were regarded as no evidence for substance misuse and were classified as no change.

Following team agreement on this coding framework, the first author then recoded all datalines using this framework, in discussion with the second author. This provided opportunities to check validity of codes against data, clarify where meaning was uncertain, and encourage reflexivity. Text was analyzed with the researchers blinded to the cause of death, except in 15 cases where the text made this explicit. After a final review of the 11 category coding framework against the full dataset, the team agreed on its conceptual coherence and did not feel there was a need to elaborate coding further.

#### Quantitative Component of Content Analysis

1. Frequencies.

Once all responses had been coded, we derived frequency counts for the 11 substance use categories to describe patterns of use by substances used. We did not include cigarette use due to small numbers, and because our research question did not directly address this.

1. Hypothesis-testing.

We imported our coded data into Stata together with variables describing socio-demographic and clinical characteristics, and characteristics of the bereavement, as collected in Part 1 of the online questionnaire.

To test the hypothesis that people bereaved by suicide, and people bereaved sudden unnatural (non-suicide) causes of death, were more likely to describe an increase in substance use than people bereaved by sudden natural causes of death we collapsed our 11 codes into a binary measure of substance use: increase in use post-bereavement vs. no increase. To separate these out by substance used, we also created two binary measures: any increase in alcohol use post-bereavement and any increase in drug use post-bereavement. Again, we did not include cigarette use.

We used descriptive statistics (counts and percentages) and chi-squared tests to describe the socio-demographic and clinical characteristics of our sample, including the following variables:

• Socio-economic status, derived from a question about own occupation (for university staff) or parental occupation (for students), using the five categories from the Office for National Statistics (ONS).
• Pre-bereavement depression using the Composite International Diagnostic Interview (CIDI) screen for lifetime depression (Robins et al., 1988), qualified by whether this was before or after the sudden bereavement, to derive a pre-exposure measure.
• Family history of psychiatric problems (including drug and alcohol problems), using responses to the question “Has anyone in your family suffered from an anxiety disorder, a depressive disorder (including postnatal depression), had drug or alcohol problems, or other psychological or emotional difficulties?

We used multivariable logistic regression to test for an association between type of bereavement and likelihood of increased substance use. We pre-selected the following socio-demographic and clinical characteristics as potential confounders of any association, based on the literature and our clinical experience: age, gender, time since bereavement, socio-economic status, pre-bereavement depression, and family history of psychiatric problems (including drug or alcohol problems). We fitted binary models using *xtlogit* commands in Stata, with HEI as random effect (Rabe-Hesketh and Skrondal, 2012), to take into to account any clustering effect at institutional level. Our statistical analyses were conducted using Stata version 15 (StataCorp, 2017).

## Results

### Participant Response

A total of 5,085 people of the 659,572 sampled responded to the questionnaire by clicking on the survey link, with 91% (*n* = 4,630) consenting to participate. Of all those consenting, 1,855 (40%) responded to the open question on substance use. Bereavement status for this group was as follows: 1,106 reported bereavement by sudden natural causes, 395 by sudden unnatural causes, and 353 by suicide. We excluded one case in which exposure was not specified. Our final sample for this analysis was therefore 1,854 individuals. There was no accurate way of measuring overall response to the survey, as the denominator of bereaved people could not be ascertained.

### Content Analysis: Frequency Counts

Frequency counts for the patterns alcohol or drug use identified are shown in Table 1. Just over half the sample (58%) reported no change in their use of alcohol, the large majority (85%) reported no changes in use of unprescribed drugs. These categories included those who had not used those substances previously. A minority had increased their alcohol use (33%) or their drug use (12%) at any point since the loss. A small minority (*n* = 94; 5%) of respondents reported having found it helpful to increase their alcohol use after the bereavement, of whom 24 (26%) had since reduced down to their pre-bereavement pattern of alcohol use. Only 47 (3%) of the sample reported finding it helpful to have increased their use of unprescribed drugs after the loss, of whom 8 (17%) had since reduced down to their pre-bereavement pattern of drug use.

**TABLE 1:** Frequency counts for content analysis (11 category coding).

Type of bereavement	Sudden natural mortality causes (*n* = 1,106)		Sudden unnatural mortality causes (*n* = 395)		Suicide (*n* = 354)		Total (*n* = 1,854)	
				
Coding category	Alcohol	Drugs	Alcohol	Drugs	Alcohol	Drugs	Alcohol	Drugs
No change in use n (%)	671 (61)	963 (87)	210 (53)	330 (84)	190 (54)	288 (82)	1,071 (58)	1,581 (85)
Brief temporary increase (<1 week) n (%)	4 (<1)	1 (<1)	3 (1)	0 (0)	2 (1)	0 (0)	9 (1)	1 (<1)
Stopped n (%)	18 (2)	9 (1)	7 (2)	10 (3)	9 (3)	8 (2)	34 (2)	27 (1)
Reduced n (%)	60 (6)	10 (1)	27 (7)	3 (1)	21 (6)	10 (3)	108 (6)	23 (1)
Increased (unclear if perceived as helpful or harmful) n (%)	189 (17)	64 (6)	78 (20)	21 (5)	69 (20)	24 (7)	336 (18)	109 (6)
Increased (perceived as helpful) n (%)	49 (4)	21 (2)	10 (3)	10 (3)	11 (3)	8 (2)	70 (4)	39 (2)
Increased (perceived as harmful) n (%)	7 (1)	1 (1)	4 (1)	0 (0)	2 (1)	2 (1)	13 (1)	3 (<1)
Increased (unclear if perceived as helpful or harmful) but then resumed pre-loss pattern of use n (%)	92 (8)	31 (3)	46 (12)	17 (4)	42 (12)	12 (3)	180 (10)	60 (3)
Increased (perceived as helpful) but then resumed pre-loss pattern of use n (%)	11 (1)	4 (<1)	8 (2)	3 (1)	5 (1)	1 (<1)	24 (1)	8 (<1)
Increased (unclear if perceived as helpful or harmful) but then stopped n (%)	2 (<1)	1 (<1)	0 (0)	0 (0)	0 (0)	0 (0)	2 (<1)	1 (<1)
Unable to classify (%)	3 (<1)	0 (0)	2 (1)	0 (0)	2 (1)	0 (0)	7 (<1)	0 (0)
No mention of illicit drug use n (%)	0 (0)	1 (<1)	0 (0)	1 (<1)	0 (0)	0 (0)	0 (0)	2 (<1)
**Summary measures of above**								
No change (excluding initial week) n (%)	675 (61)	964 (87)	213 (54)	330 (84)	192 (54)	288 (82)	1,080 (58)	1,582 (85)
Increased at any point beyond initial week n (%)	350 (32)	122 (11)	146 (37)	51 (13)	129 (37)	47 (13)	625 (33)	220 (12)
Reduced or stopped n (%)	78 (7)	19 (2)	34 (9)	13 (3)	30 (9)	18 (5)	142 (8)	50 (3)
Total	1,106 (100)	1,106 (100)	395 (100)	395 (100)	354 (100)	354 (100)	1,854 (100)	1,854 (100)

A small proportion had completely stopped their use of alcohol (2%) or drugs (1%). Quotes to illustrate responses coded in each category are given in Table 2, edited only for spelling errors.

**TABLE 2:** Examples of free text responses by coding category.

Code	Example quote (type of bereavement in brackets)
1 No change in use	“It has not altered my drinking habits or my used of drugs. I would say that I am a social drinker and still am. I used to be also a social drug user as well, mainly weed, but I have not taken drugs in a very long time” (sudden natural causes).
2 Brief temporary increase (<1 week)	“It hasn’t affected me in the long term, but the night I found out what had happened I drank to try and forget about it, but instead I became more miserable and ended up going home in a ridiculous, drunken state” (suicide).
3 Stopped	“Stopped drinking alcohol immediate aftermath of bereavement as felt numb, alcohol appeared to have no effect on me and I was afraid to drink too much as I know alcohol is a depressant (I was depressed enough!)” (sudden unnatural causes)
4 Reduced	“I was more dedicated to not using drink or drugs for fear any use would tempt me to use them to drown my sorrows and lead to addiction” (suicide).
5 Increased (unclear if perceived as helpful or harmful)	“I have hit alcohol quite hard at certain times since the bereavement, sometimes I can go weeks without a single drop, but other times I could get drunk 5 days in a week” (sudden natural causes).
6 Increased (perceived as helpful)	“I definitely drink to numb myself and physically relax, and I would say I’ve drunk more heavily since Dad died” (sudden natural causes).
7 Increased (perceived as harmful)	“My drinking increased significantly and was self-destructive. Even though it was a death from natural causes I carried a lot of guilt about being alive when he wasn’t and this carried on to my 30th birthday (when I became older then he was) but I had managed to get a hold of and sort my drinking out a few months before this time. I never indulged in drugs” (sudden natural causes).
8 Increased (unclear if perceived as helpful or harmful) but then resumed pre-loss pattern of use	“For the couple of months following his death, I definitely drank more, but I think it was more a case of making sure I was out and busy all the time. Now my drinking habits are pretty much as they were” (sudden natural causes). “I drank a fair bit in the first year, whilst going out partying hard and trying to find oblivion. I now socially drink only” (suicide).
9 Increased (perceived as helpful) but then resumed pre-loss pattern of use	“In the first month following his death I was drinking excessively and smoking marijuana a lot to try and numb myself from the reality of what had happened” (sudden unnatural causes).
10 Increased (unclear if perceived as helpful or harmful) but then stopped	“After the bereavement I drunk a lot of alcohol and went out a lot but now I don’t drink at all” (sudden unnatural causes)
11 Unable to classify	“I feel guilty about drinking” (sudden unnatural causes) “Whenever I drink too much I become depressed and cry a lot about the death” (sudden natural causes).

Table 1 also presents three basic summary measures, namely: no impact of bereavement on substance use (except initial week); increase in use (at any point beyond the first week after the loss); and decreasing or stopping use. We found no significant between-group differences on these categories using simple chi-squared tests for alcohol (*p* = 0.973) but a borderline significant group difference for drug use (*p* = 0.046).

Although not presented in this table, and not specifically probed in the question, 39 respondents (2%) mentioned a change in their smoking habits. Of these, five had stopped smoking since the bereavement, three had cut down on smoking, and 31 had increased their smoking (for whom this had been temporary in eight cases).

### Participant Characteristics

The characteristics of the sample of *n* = 1,854 individuals included in the current analysis are presented in Table 3.

**TABLE 3:** Sociodemographic characteristics of survey respondents (*n* = 1,854).

Bereavement exposure	Sudden natural causes (*n* = 1,106) n (%)	Sudden unnatural causes (*n* = 395) n (%)	Suicide (*n* = 353) n (%)	Total (*n* = 1,854) n (%)	*p*-value^a^
**Socio-demographic characteristics**					
**Gender^†^**					
Male	217 (20)	68 (17)	67 (19)	352 (19)	0.579
Female	889 (80)	327 (83)	286 (81)	1,502 (81)	
Missing	0	0	0	0	
**Age of participant (years)^†^**					
Mean (SD)	25.5 (6.4)	25.8 (6.4)	25.6 (6.0)	25.6 (6.3)	**0.006**
Aged 18–21	412 (37)	133 (34)	112 (32)	657 (35)	0.119
Aged 22–40	694 (63)	262 (66)	241 (68)	1,197 (65)	
**Relationship status**					
Single	759 (69)	277 (70)	231 (65)	1,267 (68)	0.132
Within a relationship	347 (31)	116 (29)	121 (34)	584 (32)	
Missing	0 (0)	2 (1)	1 (<1)	3 (<1)	
**Self-defined ethnicity**					
White	994 (90)	362 (92)	323 (92)	1,679 (91)	0.242
Non-white	112 (10)	32 (8)	30 (9)	174 (9)	
Missing					
**Socio-economic status^†^**^b^					
Social classes 1.1 and 1.2	680 (62)	252 (64)	222 (63)	1,154 (62)	0.802
Social classes 3 –7 and 9	402 (36)	132 (33)	124 (35)	658 (36)	
Missing	24 (2)	11 (3)	7 (2)	42 (2)	
**Educational status**					
Attained maximum A level equivalent	473 (43)	156 (40)	128 (36)	757 (41)	0.187
Attained degree level or above	630 (57)	238 (60)	225 (64)	1,903 (59)	
Missing	3 (<1)	1 (<1)	0 (0)	4 (<1)	

**Clinical characteristics**					

**Pre-bereavement depression**^†c^					
Yes	230 (21)	72 (18)	92 (26)	384 (21)	**0.037**
No	876 (79)	322 (82)	260 (74)	1,458 (79)	
Missing	0 (0)	1 (<1)	1 (<1)	2 (<1)	
**Family history of any psychiatric problems (including drug and alcohol problems)^†^**					
Yes	727 (67)	270 (69)	255 (72)	1,262 (68)	0.287
No	368 (33)	124 (31)	98 (28)	590 (32)	
Missing	1 (<1)	1 (<1)	0 (0)	2 (<1)	

**Characteristics of the bereavement**					

**Kinship to the deceased**					
Blood-related	168 (15)	199 (50)	170 (48)	537 (29)	**<0.001**
Non blood-related	934 (85)	193 (49)	183 (52)	1,310 (71)	
Missing	4 (<1)	3 (1)	0 (0)	7 (<1)	
**Years since bereavement^†^**					
Mean (SD)	4.8 (5.3)	5.1 (5.2)	5.1 (5.0)	4.9 (5.2)	0.066
less than2 years	376 (34)	106 (27)	98 (28)	580 (31)	**0.009**
Over 2 years	730 (66)	289 (73)	255 (72)	1,274 (69)	
Missing	0	0	0	0	

*^†^Covariates included in adjusted model. ^a^p-values for univariate associations of characteristics with loneliness scores. ^b^Socio-economic status using the five categories from UK Office for National Statistics. ^c^Measured using CIDI screen for depression. p-values in bold indicate those below the threshold for statistical significance.*

The sample was primarily female (81%), and of white ethnicity (91%), with the majority representing social classes 1 and 2 (62%) and educated to degree level or above (59%). The age range of respondents was 18–40 years as per eligibility criteria (mean = 25.6; *SD* = 6.3). Length of time since the index bereavement ranged from 1 day to 28 years (mean = 4.9 years; *SD* = 5.2; IQR = 6 years). The majority of the sample (68%) reported a family history of mental health problems, including drug and alcohol problems. The majority of those bereaved by sudden natural mortality causes (85%) reported the death of a relative, but those bereaved by sudden unnatural causes and suicide were split equally between those bereaved by the death of a relative or a non-relative. Overall 21% of the sample had a personal history of depression pre-dating the bereavement, but this was significantly more common in those bereaved by suicide (26%) than in those bereaved by sudden natural losses (21%) or other unnatural losses (18%).

### Association Between Bereavement Exposure and Changes in Substance Use

Our multivariable logistic regression found that, compared with people bereaved by sudden natural causes, both those bereaved by suicide and those bereaved by sudden unnatural causes were significantly more likely to describe increased use of alcohol or unprescribed drugs post-bereavement (Table 4). This was the case in unadjusted and adjusted analyses, although this was of borderline significance in people bereaved by suicide (*p* = 0.049).

**TABLE 4:** Associations between substance use and type of bereavement.

Exposure to bereavement by:	Sudden natural deaths (*n* = 1,106)		Sudden unnatural deaths (*n* = 395)			Suicide (*n* = 353)			Total (*n* = 1,854)
				
Outcome	Prevalence n (%)	Odds ratio	Prevalence n (%)	Prevalence n (%)	Adjusted^*a*^ odds ratio (95% CI)	Prevalence n (%)	Unadjusted odds ratio (95% CI)	Adjusted^*a*^ odds ratio (95% CI)	Prevalence n (%)
Any increase in substance use post-bereavement	394 (36)	1	160 (41)	700 (38)	1.32 (1.03–1.68) **(*p* = 0.026)**	146 (41)	1.30 (1.01–1.67) **(*p* = 0.038)**	1.29 (1.00–1.66) **(*p* = 0.049)**	700 (38)
Any increase in alcohol use post–bereavement	350 (32)	1	146 (37)	625 (34)	1.35 (1.05–1.73) **(*p* = 0.017)**	129 (37)	1.26 (0.97–1.62) (*p* = 0.081)	1.25 (0.96–1.61) (*p* = 0.096)	625 (34)
Any increase in drug use post-bereavement	122 (11)	1	51 (13)	220 (12)	1.31 (0.91–1.88) (*p* = 0.147)	47 (13)	1.30 (0.90–1.87) (*p* = 0.157)	1.28 (0.88–1.86) (*p* = 0.190)	220 (12)

*^a^Adjusted for age, gender, socio-economic status, time since bereavement, pre-bereavement depression, and family history of psychiatric problems (including drug and alcohol problems); threshold for significance for all models was p = 0.05. p-values in bold indicate those below the threshold for statistical significance.*

When separated out into alcohol and drug use, only the group bereaved by sudden unnatural causes were significantly more likely to describe increased alcohol use post-bereavement. There was no evidence for between-group differences in post-bereavement use of unprescribed drugs. However, these sub-group analyses may have been underpowered to detect any differences, and the magnitudes of the odds ratios for these individual measures were very similar. Although we did not directly compare bereavement by suicide and non-suicide unnatural causes, in all models confidence intervals for the odds ratios for each group overlapped, suggesting no differences.

## Discussion

### Main Findings

The findings of our content analysis suggest that in a sample of people who had experienced sudden bereavement, the majority had not changed their pattern of drug or alcohol use. This sample had been bereaved for an average of 5 years, capturing both short-term and longer-term effects over a period of grieving. However, a third of the bereaved sample had increased their alcohol use at some point, whilst a fifth had increased their use of unprescribed drugs. Our content analysis identified that an awareness of benefits or harms was apparent in some respondents’ accounts of any changes in their patterns of use. A perception of benefits was more common than that of harms, although only a small number expressed such judgments.

We found that people bereaved by suicide or other unnatural deaths were significantly more likely than those bereaved by sudden natural deaths to describe an increase in substance use. However, this finding was of borderline significance for people bereaved by suicide, and our analyses may have lacked power when separating out drugs and alcohol use. An increase in substance use after bereavement by suicide or other unnatural losses is in keeping with the clinical understanding of unnatural losses as more traumatic for the bereaved than deaths due to natural causes, and the potential for the bereaved to cope with the loss by using drugs or alcohol, even if transiently. It is possible that people bereaved by suicide and other unnatural causes are more likely to respond behaviorally to their loss by using substances to cope. This may meet a need to escape from the work of grief, to palliate symptoms of depression or anxiety, or to find a release. Such substance use may be within safe limits, but in some cases likely to be hazardous (Public Health England, 2016).

It is also possible that people bereaved by suicide or other unnatural deaths have genetic similarities to the people they grieve, for whom substance use may have contributed to the death, and therefore a genetic vulnerability to substance misuse after a traumatic life event. Individuals diagnosed with alcohol dependence have a greater risk of death by unnatural causes, whether due to suicide, homicide or accidental death (Markkula et al., 2012). They leave behind partners and friends who are genetically similar, based on theories of assortative mating (Agerbo, 2005) and assortative relating (Joiner, 2003) and therefore also at risk of substance misuse. Whether bereavement by suicide or other unnatural death triggers substance use in those with or without genetic vulnerabilities, it is important that we are aware of these needs. Our findings suggest that people bereaved by suicide or other unnatural deaths may have a need for primary care monitoring of substance use, to identify any emergent harmful use and offer support to address this. They also suggest that clinicians should be mindful of the perceived beneficial effects of substance use as part of the normal process of grief, and balance this against the potential for harmful effects.

### Results in the Context of Other Studies

No other studies have used mixed methods to investigate the impact of bereavement on self-perceived substance misuse in a sample of young adults in Britain bereaved suddenly by the death of a family member or a friend. In relation to existing quantitative research, our findings using self-reported accounts conflict with studies using diagnostic categories, perhaps due to the greater detail we explored. A Canadian registry-based study used routine clinical data to identify ICD-9 and ICD-10 codes for alcohol abuse or dependence in parents bereaved by the suicide or the motor vehicle death of a child (Bolton et al., 2013). This found that each group’s probability of drug abuse/dependence or of alcohol abuse/dependence did not significantly increase after the loss (Bolton et al., 2013). Although this was methodologically more stringent in its use of strict thresholds for diagnosis, it did not account for a range of behaviors within and beyond those diagnostic criteria, as ours did. One other British study investigating alcohol use after suicide bereavement was an uncontrolled study of 85 people bereaved by the suicide of an older adult using semi-structured interviews. This found that alcohol intake had increased in 14%, although the measure used was not specified. Self-reported consumption in terms of units of alcohol indicated that 7% were drinking at hazardous levels (Harwood et al., 2002). The same study also collected data on change in alcohol use in the group bereaved by suicide and a control group of those bereaved by natural causes, but did not report these findings (Harwood et al., 2002), and those data are no longer available.

In relation to other qualitative research, our own thematic analyses of these data specifically for people bereaved by suicide (Eng et al., 2019), and non-suicide unnatural deaths (Drabwell et al., 2020), identified that for both groups struggles over control with alcohol or drugs were prominent and many used substances for specific ends. For those bereaved by non-suicide unnatural causes these ends included gaining a release, escaping reality, and achieving emotional openness (Drabwell et al., 2020). For those bereaved by suicide, they included coping with overwhelming emotions, and honoring the memory of the deceased (Eng et al., 2019). For both groups there was a clear awareness of the harmful effects balanced against these perceived benefits, which for many drove efforts to control their use (Eng et al., 2019; Drabwell et al., 2020). A thematic analysis of interviews with 57 young British men aged 19–35 who had been bereaved by non-suicide unnatural causes found many similar themes (Creighton et al., 2015). In this sample many men explained that they drank to anesthetize themselves from the pain, whilst others used alcohol to release feelings of despair. Many of the older participants felt that the positive effects of alcohol outweighed the negative effects over the short-term, whilst also recognizing negative effects in longer-term use, whereby ingrained habits could lead to risk-taking and dependence (Creighton et al., 2015). Together this body of work suggests that an understanding of the perceived benefits and risks is important when discussing with a bereaved person their use of alcohol or drugs as part of their grief. Their needs for substance misuse support should therefore be considered and discussed in the context of the balance of risks and benefits they perceive.

### Strengths and Limitations

We believe this to have been the largest-scale qualitative study comparing self-reported use of alcohol and unprescribed drugs after sudden bereavement, and comparing suicide to non-suicide losses. Other studies using national registries have achieved larger sample sizes, but used recorded diagnoses of substance misuse or dependence rather than self-reported changes in patterns of use, and these may result in under-ascertainment of harmful or hazardous substance use. Although our categories had no diagnostic validity, they had face validity from the perspective of a bereaved person and emphasized any potential benefits perceived as well as perceived harmful consequences. Our models were not adjusted for pre-bereavement substance misuse, due to the lack of a validated measure in our survey, but our outcomes were based on a perceived change in use rather than diagnostic criteria *per se*. Covariates were chosen for our model *a priori*, using a theory-driven approach based on the scientific literature. However, we acknowledge the possibility of residual confounding. Our sample comprised specific subgroups warranting closer investigation, including those who had never used alcohol or drugs as part of their coping repertoire, those using alcohol and prescribed drugs within safe limits, and those using them at hazardous levels. These groups are likely to have very different cognitive profiles, but again we were not able to explore these using our data.

We used a novel but systematic approach to test a specific research question. Face validity and external validity were enhanced by using minimal dimensions during coding, namely a perceived increase or decrease in use, and any perception of the change being helpful or harmful. The advantage of using content analysis was that we were able to organize complex free text data into clear themes and relate these to quantitative variables of clinical interest. Use of an anonymous online questionnaire for this sensitive topic enhanced disclosure (Joinson, 2001) and reduced the potential for social desirability bias effects (Joinson, 1999) but it remains possible that denial or shame may have resulted in under-reporting of substance use. This is despite accepting attitudes toward alcohol in some cultures (Sudhinaraset et al., 2016), particularly among young British people (Cooke et al., 2019). The wording of the question used may have resulted in the under-reporting of legal psychoactive drug use, which has become more prevalent in this age group in Britain (Bowden-Jones, 2013), and of changes in tobacco use, which were reported unprompted by 2% of the sample. Given the age group and student status of many respondents it was possible that changes in substance use were influenced more by peer groups or educational transitions than the bereavement itself. However, given the nature of the data collection it was not possible to disentangle this further.

Our sample was derived from higher education institutions in the UK, with a bias toward those in higher social classes. This and the non-response from men suggests that the sample may not be generalizable to all young bereaved adults in the UK, but instead may have more resonance to female, white, highly-educated groups. However, this approach was preferable to that of recruiting a help-seeking sample, which risked introducing selection biases. Our sampling method also allowed access to a large community sample of young adults who tend not to be represented in health research. As only 40% of those consenting to participate in the wider survey answered the question on substance use, this is likely to have introduced a response bias from those with the time to provide more detailed responses and/or those with potentially the strongest views on this issue. This again limits the generalizability of our findings. Online data collection meant that we were unable to probe the motivations for respondents’ patterns of substance use, as might have been possible in an interview study. Although our data were collected in 2010, Global Burden of Disease data show that alcohol remained the leading risk factor for ill-health, disability and early mortality among people aged 15–49 years over the period 1990–2013 (Forouzanfar et al., 2015). No other study to our knowledge has collected similar data, so this constitutes the best available evidence. Our thematic analyses of data for people bereaved by suicide (Eng et al., 2019), and by accidental death (Drabwell et al., 2020), were conducted with lead researchers blind to the coding used in this study, and provide further insights into the reasons for underlying behavior change in this sample.

### Clinical, Policy, and Research Implications

Our findings provide clinically valuable information for primary care practitioners and bereavement counselors encountering bereaved young adults in clinical settings. A third of our sample had increased their alcohol use, whilst a fifth had increased their drug use. This appears to be one method of coping with sudden bereavement, but places them at theoretical risk of harm, with the possibility of short-term harms (through disinhibition, vulnerability to violence, and toxicity) as well as longer-term organ damage if use becomes hazardous or dependent. Substance misuse is likely to be a correlate of other putative risk factors for early mortality after bereavement, such as psychological distress, loneliness, and loss of a confidant(e), as well as changes in social ties, living arrangements, and economic support (Byrne et al., 1999; Stroebe et al., 2007). Use of drugs or alcohol should therefore be viewed and discussed in this wider psychosocial context. Overall 2% of respondents stopped using alcohol completely, and 1% stopped using drugs. This is a small but clinically important behavioral response to sudden bereavement, with potential health benefits, and likely explanations are as follows. Our previous thematic analysis of qualitative data from the sub-sample of those bereaved by suicide identified a tendency in some people to respond to the loss by developing their own safeguards against suicide (Pitman et al., 2017). These included avoiding all medications for fear of accidental overdose. The attitudes underlying complete cessation of alcohol or drugs after sudden bereavement in the current study might therefore relate to a desire to protect oneself from a similar death, whether due to sudden cardiac causes, accidental death, or suicide. Our more in-depth thematic analyses of this dataset also suggested that some people bereaved by unnatural causes stopped using drugs or alcohol because alcohol made them depressed (Drabwell et al., 2020), alcohol made it harder for them to control their emotions (Eng et al., 2019), or because drugs or alcohol were thought to have contributed to the death (Eng et al., 2019). The attitudes underlying complete cessation of alcohol or drugs after sudden bereavement across a broader sample of people bereaved by sudden natural or unnatural causes are likely to relate to a desire to protect oneself from a similar death, and to avoid the negative effects.

Our results suggest that all young adults exposed to sudden bereavement should be screened for substance use, particularly after bereavements due to suicide and other unnatural causes, as part of an inquiry about methods of coping. Not all will be in touch with bereavement counselors, particularly as provision of bereavement support varies greatly geographically. However, some will consult their primary care teams, perhaps for the physical and mental health problems that are more common after sudden bereavement (Erlangsen et al., 2017). Bereavement counselors and primary care practitioners should inquire non-judgmentally about current substance misuse, and explore whether the bereaved person would like any help to cut down. Targeting such screening at those bereaved by suicide and other unnatural causes would be indicated given the findings of this study, but should not be punitive (Eng et al., 2019; Drabwell et al., 2020). Given that bereavement services are often disconnected from health services, and bereavement counselors may not feel skilled to screen for substance use where they suspect it, appropriate liaison between the two services would be helpful. Our separate more fine-grained analyses of free text responses for people bereaved by suicide (Eng et al., 2019) and by other sudden unnatural deaths (Drabwell et al., 2020) provide clinicians with a more nuanced understanding of the reasons for drug or alcohol use. An awareness of these reasons would help clinicians broach the issue in a more acceptable way, increasing the likelihood of successful intervention. It would also help concerned friends and relatives know how to offer support without appearing judgmental.

## Conclusion

Among young adults in the UK who experience the sudden death of a friend or relative, a third describe an increase in their use of alcohol or unprescribed drugs, whether transiently or permanently. This is significantly more likely in people bereaved by suicide or other unnatural deaths. Despite the perceived advantages in helping them cope with grief, substance use carries a risk of short-term harm and permanent organ damage, and may not be identified by professionals offering bereavement support. Our coding identified that some bereaved people perceive benefits in using drugs or alcohol as part of their adjustment to the loss, particularly in the immediate aftermath, suggesting that a non-judgmental approach from professionals is important. A discussion of coping mechanisms at different stages after a traumatic loss may help identify those who are relying on drugs or alcohol more than they are comfortable with, or more than is safe, and who might need extra support.

## Data Availability Statement

The dataset generated for this article is not publically available due to the threat to confidentiality. However, requests for collaboration to analyze these data can be made to the lead author, subject to departmental review of proposal and honorary contract.

## Ethics Statement

The study was approved by the UCL Research Ethics Committee in 2010 (ref: 1975/002). The patients/participants provided their written informed consent to participate in this study.

## Author Contributions

AP, DO, and MK conceived and designed the study and the recruitment strategy. AP, DO, MK, and FS contributed to questionnaire design. AP recruited participants, managed the survey, collected data, developed the coding framework, ran the regressions, drafted the manuscript with input from all authors, had full access to all the data in the study, takes responsibility for the integrity of the data, and was the guarantor. AP and FS independently coded data.

## Conflict of Interest

The authors declare that the research was conducted in the absence of any commercial or financial relationships that could be construed as a potential conflict of interest.
